# Supplementary material for: From sequence to enzyme mechanism using multi-label machine learning
Source: BMC Bioinformatics. 2014 May 19;15:150. doi: 10.1186/1471-2105-15-150 (PMC4229970; doi:10.1186/1471-2105-15-150)
Supplement: Additional file 2 — Java code of ml2db. Additional file ml2db_code.tar.gz contains the Java source code to run the multi-label machine learning experiments and save the results to database. The code’s Javadoc is included. [file 1471-2105-15-150-S2.zip › additional file 2/ml2db/utils/doc/index-files/index-2.html]

B-Index


JavaScript is disabled on your browser.


- Overview
- Package
- Class
- Use
- Tree
- Deprecated
- Index
- Help

- Prev Letter
- Next Letter

- Frames
- No Frames

- All Classes

A B C D E F G H I J K L M N O P Q R S T U V W X Y 


## B

backQuoteChars(String) - Static method in class uk.ac.standrews.utils.main.stringutils.StringUtils
:   Converts carriage returns and new lines in a string into \r and \n.

BIG\_PRIME - Static variable in class edu.cornell.lassp.houle.RngPack.Ranmar
:   The 46,009,220nd prime number, he largest prime less than
    9\*10^8^.

BOOLEAN\_SQL\_DATATYPE - Static variable in class uk.ac.standrews.utils.main.database.DbUtils


BooleanArrayPool - Class in uk.ac.standrews.utils.main
:   Class

BooleanArrayPool(int) - Constructor for class uk.ac.standrews.utils.main.BooleanArrayPool


booleanArrayToString(boolean[]) - Static method in class uk.ac.standrews.utils.main.collectionutils.ArrayUtils


booleanArrayToString(boolean[]) - Static method in class uk.ac.standrews.utils.main.stringutils.StringUtils


booleanTo01String(Boolean) - Static method in class uk.ac.standrews.utils.main.stringutils.StringUtils
:   Returns "1" if Boolean.TRUE, "0" if Boolean.FALSE

breakApplication(Object) - Static method in class uk.ac.standrews.utils.main.SystemUtils

A B C D E F G H I J K L M N O P Q R S T U V W X Y

- Overview
- Package
- Class
- Use
- Tree
- Deprecated
- Index
- Help

- Prev Letter
- Next Letter

- Frames
- No Frames

- All Classes
